# On Some Extension of Intuitionistic Fuzzy Synthetic Measures for Two Reference Points and Entropy Weights

**DOI:** 10.3390/e24081081

**Published:** 2022-08-05

**Authors:** Ewa Roszkowska, Bartłomiej Jefmański, Marta Kusterka-Jefmańska

**Affiliations:** 1 Faculty of Computer Science, Bialystok University of Technology, Wiejska 45A, 15-351 Bialystok, Poland; 2 Department of Econometrics and Computer Science, Wroclaw University of Economics and Business, 53-345 Wrocław, Poland; 3 Department of Quality and Environmental Management, Wroclaw University of Economics and Business, 53-345 Wrocław, Poland

**Keywords:** fuzzy multi-criteria decision making, fuzzy synthetic measure, intuitionistic fuzzy sets, entropy weights, ideal-point, anti-ideal point

## Abstract

In this paper, a novel Double Intuitionistic Fuzzy Synthetic Measure (DIFSM), based on intuitionistic fuzzy values for handling multi-criteria decision-making problems used to rank alternatives, is presented. In the studies, intuitionistic fuzzy sets (IFSs) represented uncertain, imprecise information or human judgment. The intuitionistic fuzzy sets can also reflect the approval, rejection, and hesitation of decision-makers. The degrees of satisfiability and non-satisfiability and uncertainty of each alternative with respect to a set of criteria are described by membership functions, non-membership functions, and hesitancy indexes, respectively. The aggregation algorithm DIFSM is inspired by Hellwig’s method based on two reference points: ideal point (pattern) and anti-ideal point (anti-pattern), measuring distances between the alternative and ideal point and distance between the ideal and anti-ideal point. The proposed methods take into consideration the entropy-based weights of criteria. An illustrative example is given to demonstrate the practicality and effectiveness of the proposed approach. Additionally, the comparative analysis results, using the DIFSM and the Intuitionistic Fuzzy TOPSIS-based framework, are presented.

## 1. Introduction

Many multi-criteria decision problems involve decision making under uncertainty, with incomplete or imprecise information. The methodologies for dealing with this problem have been developed in various directions [1,2]. The decision-making problems with uncertain information about the criteria can be handled with the help of fuzzy sets [3], intuitionistic fuzzy sets [4], and interval intuitionistic fuzzy sets [5,6], among others. The concept of the intuitionistic fuzzy set (IFS) is based on the simultaneous consideration of three aspects of information: membership (μ), non-membership $\left(\nu_{\;}\right),$ and hesitation degree or degree of uncertainty (π) [7]. The intuitionistic fuzzy multi-criteria or group intuitionistic fuzzy multi-criteria methods have been widely used in practical problems such as supplier selection [8,9], mobile phone selection [10], personnel selection [11], sustainable energy management [12], analysis of socio-economic phenomena in survey data [13,14], evaluations of negotiation offers [15], and medical diagnostics [16], among others.

This paper aims to develop a new intuitionistic fuzzy multi-criteria decision-making technique, Double Intuitionistic Fuzzy Synthetic Measure (DIFSM), for solving decision problems under uncertainty in which the criteria weights are unknown. The aggregation algorithm DIFSM is inspired by Hellwig’s method, based on two reference points: ideal solution (pattern) and anti-ideal solution (anti-pattern), which are used to integrate the information [17]. The ideal and anti-ideal points are determined, and the distances between each alternative and the ideal point are calculated. The weights are obtained using an entropy measure that is more objective and does not require the decision-maker to specify the importance of the criteria. The weighted Euclidean distances for intuitionistic fuzzy sets between alternatives and the ideal point were calculated. The distance between ideal and anti-ideal points is used to normalize the aggregation-measure DIFSM. Finally, the alternatives are ranked by comparing the relative closeness of the ideal point.

The synthetic-measure DIFSM proposed in this paper is an extension of the IFSM measure, which was originally used to analyze complex socio-economic phenomena described by ordinal data. To draw attention to the wider possibilities of the application of both measures, the DIFSM algorithm was presented as a multi-criteria decision-making method. Therefore, a numerical example is given to illustrate the feasibility and effectiveness of the proposed method, by solving a multi-criteria decision problem of the choice of air-conditioning system installed in a library.

This paper is organized as follows. The classical Hellwig method based on two reference points is presented in Section 2. The definition and properties of intuitionistic fuzzy sets are briefly introduced in Section 3. Next, a multicriteria decision-making method based on intuitionistic fuzzy sets DIFSM is proposed in Section 4. A numerical example and short conclusion are given in Section 4 and Section 5, respectively.

## 2. The Hellwig Method Based on Two Reference Points

The Hellwig method was originally proposed in 1968 as a taxonomic method for international comparisons of the economic development of countries [18] and was popularized in the international literature in 1972 with the realization of the UNESCO research project on human resource indicators for less-developed countries [19,20]. The construction of Hellwig synthetic measure is based on the distances of objects from the abstract pattern of economic development (ideal point).

The areas of application of the classic Hellwig method and its modifications include the following: an evaluation of the competitive balance of the Italian Football League according to the taxonomic approach [21], determination of the differences in socio-economic development among 28 EU economies [22], analyzing differences in agricultural performance across the EU countries [23], evaluation of the implementation of the Europe 2020 strategy in education across EU countries [24], ranking of EU countries in terms of the value of environmental governance indicators [25], assessments of the level of agricultural development in 28 countries of the EU [26], assessments of the socio-economic development of rural Wielkopolska in Poland [27], recognizing economic types of agriculture and rural areas [28], evaluation of the subjective quality of life of residents from 11 communes of the Kraina Łęgów Odrzańskich region in Poland [13], evaluation and rank ordering the multi-issue negotiation offers [15], and assessment of the subjective quality of life of inhabitants from selected communes in Poland [29], among others. 

The less-known variant of the classical Hellwig method in the aggregation procedure takes into consideration both pattern (ideal point) and anti-pattern (anti-ideal point) of development [17]. The distance between each object (alternative) and the pattern is compared with the distance between the pattern and the anti-pattern. This variant of the Hellwig method is similar to the TOPSIS (the Technique for Order of Preference by Similarity to Ideal Solution) presented by Hwang and Yoon [30,31]. Both methods are based on two reference points and the distances of alternatives from these points, but differ in the aggregation procedure. TOPSIS is based on the concept that the chosen alternative should have the shortest distance from the positive ideal solution (ideal point) and the longest distance from the negative ideal solution (anti-ideal point).

This variant of the Hellwig method was applied to evaluate the economic efficiency of small- and medium-sized manufacturing enterprises in districts of Wielkopolska province [32,33], assess changes in population ageing in regions of the V4 countries [34], and measure social cohesion at the province level in Poland [35]. 

Here, we presented both variants of the classical Hellwig method [17,18].

Let $O=\left\{O_{1},O_{2},\ldots ,O_{m}\right\}$ i=1,2,…,m be the set of objects under assessment and $X=\left\{X_{1},X_{2},\ldots ,X_{n}\right\}$ j=1,2,…,n the set of variables constituting a complex phenomenon. It should also be adopted that *P* and *N* are the sets of stimulating (positive) and destimulating (negative) variables, respectively, influencing the complex phenomenon (X=P∪N). Hellwig’s method consists of the following steps [18]:

**Step 1.** Defining the data matrix:

(1) $$D=\left[\begin{array}{cccc} x_{11} & x_{12} & \cdots & x_{1n}\\ x_{21} & x_{22} & \cdots & x_{2n}\\ \vdots & \vdots & \ddots & \vdots \\ x_{m1} & x_{m2} & \cdots & x_{mn} \end{array}\right]$$

where *x_ij_* is the assessment of *i*-th object with respect to the *j*-th variable (i=1,2,…,m; j=1,2,…,n).

**Step 2.** Determining the normalized data matrix:

(2) $$Z=\left[\begin{array}{cccc} z_{11} & z_{12} & \cdots & z_{1n}\\ z_{21} & z_{22} & \cdots & z_{2n}\\ \vdots & \vdots & \ddots & \vdots \\ z_{m1} & x_{m2} & \cdots & z_{mn} \end{array}\right]$$

using the standardization formula:

(3) $$z_{ij}=\frac{x_{ij}-{\bar{x}}_{j}}{S_{j}}$$

where ${\bar{x}}_{j}=\frac{1}{m}\overset{m}{\underset{i=1}{\sum }}x_{ij}$, $S_{j}=\sqrt{\frac{1}{m}\overset{m}{\underset{i=1}{\sum }}{\left(x_{ij}-{\bar{x}}_{j}\right)}^{2}}$.

**Step 3.** Defining the pattern of development $O^{+}=\left[z_{1}^{+},z_{2}^{+},\ldots ,z_{n}^{+}\right]$ in accordance with the principle:

(4) $$z_{j}^{+}=\{\begin{array}{c} \underset{i}{\max}z_{ij}\;if\;z_{ij}\in P\\ \underset{i}{\min}z_{ij}\;if\;z_{ij}\in N \end{array}$$

**Step 4.** Calculating the distance of the i-th object from the pattern of development using the Euclidean distance:

(5) $$d_{i}^{+}=\sqrt{\sum_{j=1}^{n}{\left(z_{ij}-z_{j}^{+}\right)}^{2}}$$

**Step 5.** Calculating the synthetic measure of development for the i-th object:

(6) $$H_{i}=1-\frac{d_{i}^{+}}{d_{0}}$$

where: $d_{0}=\bar{d}+2S$, $\bar{d}=\frac{1}{m}\overset{m}{\underset{i=1}{\sum }}d_{i}^{+}$, $S=\sqrt{\frac{1}{m}\overset{m}{\underset{i=1}{\sum }}{\left(d_{i}^{+}-\bar{d}\right)}^{2}}$.

**Step 6.** Ranking the objects according to the decreasing values of $H_{i}$.

In the classical Hellwig measure based on two pattern objects, Formula (6) takes the following form:

(7) $$H_{i}=1-\frac{d_{i}^{+}}{d^{+-}}$$

where $d^{+-}=\sqrt{\overset{n}{\underset{j=1}{\sum }}{\left(z_{j}^{+}-z_{j}^{-}\right)}^{2}}$.

The synthetic measure *H_i_* usually takes the values from the interval [0, 1]. The higher the values of the measure, the less the object is away from the pattern of development.

## 3. Preliminaries on Intuitionistic Fuzzy Sets

### 3.1. The Notion of IFS

The fuzzy set theory was proposed by Zadeh in 1965 to deal with uncertainty [36]. The concept of intuitionistic fuzzy was introduced in 1986 by Atanassov [7] as an extention of fuzzy set.

**Definition** **1** ([7,37])**.** *Let X be a universe of discourse of objects. An intuitionistic fuzzy set A in X is given by:*

(8) $$A=\left\{\langle x,\mu_{A}\left(x\right),\nu_{A}\left(x\right)\rangle |x\in X\right\}$$

*where* $\mu_{A},\nu_{A}:X\to \left[0,1\right]$ *are functions with the condition for every* x∈X

(9) $$0\leq \mu_{A}\left(x\right)+\nu_{A}\left(x\right)\leq 1$$

The numbers $\mu_{A}\left(x\right)$ and $\nu_{A}\left(x\right)$ denote, respectively, the degrees of membership and non-membership of the element x∈X to the set *A*; $\pi_{A}\left(x\right)=1-\mu_{A}\left(x\right)-\nu_{A}\left(x\right)$ denote the intuitionistic fuzzy index (hesitation margin) of the element x in set *A*.

If the universe *X* contains only one element *x*, then the IFSA over *X* can be denoted as $A=\left(\mu_{A},\nu_{A}\right)$ and called an intuitionistic fuzzy value (IFV) [38,39]. Let Θ be the set of all IFVs. Clearly, intuitionistic fuzzy value (0, 1) is the largest, while (0, 1) is the smallest.

The advantages of applying intuitionistic fuzzy sets in decision-making [9,40,41] are when dealing with uncertainty, and incomplete or imprecise information. 

### 3.2. Distances and Similarity Measures between IFS

In the literature, we can find several concepts of distances or similarity measures between intuitionistic fuzzy sets [41,42,43,44,45,46,47]. The most widely used are Hamming and Euclidean distances based on two or three parameters [41]. In the paper, we used the concept of weighted Hamming and Euclidean distances proposed by Xu [46].

**Definition** **2** ([46])**.** *Let us consider two* A,B∈IFS *with membership functions* $\mu_{A}\left(x\right)$*,* $\mu_{B}\left(x\right)$ *and non-membership functions* $\nu_{A}\left(x\right)$, $\nu_{B}\left(x\right)$, *respectively*.

The weighted Euclidean distance is calculated in the following way:

(10) $$dE\left(A,B\right)=\sqrt{\frac{1}{2\;}\sum_{j=1}^{n}w_{j}[\;{\left(\mu_{A}\left(x_{j}\right)-\mu_{B}\left(x_{j}\right)\right)}^{2}+{\left(\nu_{A}\left(x_{j}\right)-\nu_{B}\left(x_{j}\right)\right)}^{2}+\left(\pi_{A}\left(x_{j}\right)-\pi_{B}\left(x_{j}\right){)}^{2}\right]}$$

The weighted Hamming distance is calculated in the following way:

(11) $$dH\left(A,B\right)=\sqrt{\frac{1}{2\;}\sum_{j=1}^{n}w_{j}\left[\;\left|\mu_{A}\left(x_{j}\right)-\mu_{B}\left(x_{j}\right)\left|+\right|\nu_{A}\left(x_{j}\right)-\nu_{B}\left(x_{j}\right)\left|+\right|\pi_{A}\left(x_{j}\right)-\pi_{B}\left(x_{j}\right)\right|\right]}$$

where $\overset{n}{\underset{j=1}{\sum }}w_{j}=1$.

This distance measure considers the membership degree, non-membership degree, and hesitation degree. The hesitation degree allows for more effective and complete expression of the lack of information in the decision-making process, and weights express the importance of criteria when Formulas (10) and (11) were applied in the multi-criteria decision-making method.

### 3.3. The Intuitionistic Fuzzy Entropy-Based Weights Method

Entropy, originally a thermodynamic unit, was later applied to information theory in 1940. The intuitionistic fuzzy entropy plays an important role in decision theory for describing the uncertainty of information. Szmidt and Kacprzyk [48] proposed some entropy measures for intuitionistic fuzzy sets by employing a geometric interpretation of intuitionistic fuzzy sets. Vlachos and Sergiagis [49] proposed another measure of intuitionistic fuzzy entropy, and revealed an intuitive mathematical connection between the notions of entropy for fuzzy sets and intuitionistic fuzzy sets. Yun Ye [50] presented intuitionistic fuzzy entropy, which is a generalized version of the fuzzy entropy in [51], and complementarity of existing entropy for intuitionistic fuzzy sets. Another intuitionistic fuzzy entropy measure was also proposed by Guo [52], Yuan and Zheng [53], Liu [54], and Khaleie and Fasanghari [55], among others.

The intuitionistic fuzzy entropy measure can be used in multi-criteria decision making for weights determination, in terms of the information provided by criteria, so-called entropy-based weights. According to the entropy theory, if the entropy for a criterion is small across alternatives, it should provide decision makers with useful information. Therefore, the criterion should be assigned a high weight; otherwise, such a criterion will be judged unimportant and should be evaluated as low weight [53,56,57].

Let us assume that the evaluation of the *i*-th alternative (i=1,2,…,m ) in terms of the *j*-th criterion (j=1,2,…,n) is expressed in the form of an intuitionistic fuzzy value $\left(\mu_{ij},\nu_{ij}\right)$, and $\pi_{ij}=1-\mu_{ij}-\nu_{ij}\;$. If the information about weight *w_j_* of the criterion *C_j_* is completely unknown, the entropy weights for determining the criteria weight can be calculated as follows [57]:

(12) $$\varepsilon \left(C_{j}\right)=-\frac{1}{mln2}\;\sum_{i=1}^{m}[\mu_{ij}\ln\mu_{ij}+\nu_{ij}\ln\nu_{ij}-\left(1-\pi_{ij}\right)ln\left(1-\pi_{ij}\right)-\pi_{ij}ln2]\;$$

where $\frac{1}{mln2}$—constant which assures $0\leq \;\varepsilon \left(C_{j}\right)\leq 1,j=1,2,\ldots ,n.$

We modify the original Hung and Chen [57] Formula (12) taking $\mu_{ij}\ln\mu_{ij}=0\;$(or $\nu_{ij}\ln\nu_{ij}=0)$ if $\mu_{ij}=0$ (or $\nu_{ij}=0).$ We can do this because $\underset{x\to 0+}{\lim}xlnx=0$.

The degree of divergence (*d_j_*) of the average information provided by the performance ratings on a criterion *C_j_* can be defined as:

(13) $$d_{j}=1-\varepsilon \left(C_{j}\right).$$

Finally, the entropy weight of the *j*-th criterion is calculated as follows:

(14) $$w_{j}=\frac{d_{j}}{\sum_{j=1}^{n}d_{j}}\;.$$

It is worth noting that the entropy-based weights are treated as more objective because they did not require decision-maker evaluation of the importance of the criteria, and are based only on the criteria values.

## 4. The Double Intuitionistic Fuzzy Synthetic Measure

In the classical Hellwig method, the evaluation of alternatives is given by crisp values. However, crisp values cannot adequately model some real-world situations, because human judgment and preference are often ambiguous and cannot be estimated exactly. Some modifications of the Hellwig method allow the ordering of objects (alternatives) characterized in terms of variables (criteria) expressed in the form of fuzzy sets [25,26,56,58] and intuitionistic fuzzy sets [13]. The synthetic measures described in [13,27] allow measuring complex phenomena based on the respondents’ opinions. The data are represented on an ordinal scale, and the object assessments may contain positive, negative, no answers, “difficult to say”, or “no opinion” answers. 

The Double Intuitionistic Fuzzy Synthetic Measure can be also applied in the analysis of complex phenomena based on survey data, but we presented DIFSM as a multi-criteria decision-making method to show a more general framework as well as the areas of application.

Let $A=\left\{A_{1},A_{2},\ldots ,A_{m}\right\}\;\left(i=1,2,\ldots ,m\right)$ be the set of alternatives and let $C=\left\{C_{1},C_{2},\ldots ,C_{n}\right\}\;\left(j=1,2,\ldots ,n\right)$ be the set of criteria under which the performance of alternatives will be evaluated. Moreover, *P* and *N* are the sets of benefit and cost criteria, respectively (C=P∪N). The evaluation of the *i*-th alternative in terms of the *j*-th criterion is expressed in the form of an intuitionistic fuzzy value $\left(\mu_{ij},\nu_{ij}\right)$, and $\pi_{ij}=1-\mu_{ij}-\nu_{ij}\;$. In this way, the *i*-th alternative *A_i_* is represented by the vector:

(15) $$A_{i}=\left[\left(\mu_{i1},\nu_{i1}\right),\ldots ,\left(\mu_{in},\nu_{in}\right)\right],$$

where *i* = 1, 2, …, *m*.

The steps of DIFSM are the following:

**Step 1.** Determining the Intuitionistic Fuzzy Decision Matrix.

(16) $$D=\left[\begin{array}{cccc} \left(\mu_{11},\;v_{11}\right) & \left(\mu_{12},\;v_{12}\right) & \cdots & \left(\mu_{1n},\;v_{1n}\right)\\ \left(\mu_{21},\;v_{21}\right) & \left(\mu_{22},\;v_{22}\right) & \cdots & \left(\mu_{2n},\;v_{i2n}\right)\\ \vdots & \vdots & \ddots & \vdots \\ \left(\mu_{m1},\;v_{m1}\right) & \left(\mu_{m2},\;v_{m2}\right) & \cdots & \left(\mu_{mn},\;v_{mn}\right) \end{array}\right]\;\;\;$$

where $\left(\mu_{ij},\nu_{ij}\right)$ is the intuitionistic fuzzy evaluation of the *i*-th alternative in terms of the *j*-th criterion.

**Step 2.** Defining the intuitionistic fuzzy ideal point ${\mathrm{IFI}}^{+}=\left[\left(\mu_{1}^{+},\;\nu_{1}^{+}\right),\ldots ,\left(\mu_{n}^{+},\;\nu_{n}^{+}\right)\right]$ based on max and min values following the principle:

(17) $$\left(\mu_{j}^{+},\;\nu_{j}^{+}\right)=\{\begin{array}{c} \left(\underset{i}{max}\mu_{ij},\underset{i}{min}\nu_{ij}\right)\;\mathrm{and}\;\pi_{j}^{+}=1-\left(\underset{i}{max}\mu_{ij},\underset{i}{min}\nu_{ij}\right)\;if\;j\text{-}\mathrm{th}\;\mathrm{criterion}\in P\\ \left(\underset{i}{min}\mu_{ij},\underset{i}{max}\nu_{ij}\right)\;\mathrm{and}\;\pi_{j}^{+}=1-\left(\underset{i}{min}\mu_{ij},\underset{i}{max}\nu_{ij}\right)\;if\;j\text{-}\mathrm{th}\;\mathrm{criterion}\in N \end{array}$$

**Step 3.** Defining the intuitionistic fuzzy anti-ideal point ${\mathrm{IFAI}}^{-}=\left[\left(\mu_{1}^{-},\;\nu_{1}^{-}\right),\ldots ,\left(\mu_{n}^{-},\;\nu_{n}^{-}\right)\right]$ based on max and min values following the principle:

(18) $$(\mu_{j}^{-},\;\nu_{j}^{-})=\{\begin{array}{c} \left(\underset{i}{max}\mu_{ij},\underset{i}{min}\nu_{ij}\right)\;\mathrm{and}\;\pi_{j}^{-}=1-\left(\underset{i}{max}\mu_{ij},\underset{i}{min}\nu_{ij}\right)\;if\;j\text{-}\mathrm{th}\;\mathrm{criterion}\in N\\ \left(\underset{i}{min}\mu_{ij},\underset{i}{max}\nu_{ij}\right)\;\mathrm{and}\;\pi_{j}^{-}=1-\left(\underset{i}{min}\mu_{ij},\underset{i}{max}\nu_{ij}\right)\;if\;j\text{-}\mathrm{th}\;\mathrm{criterion}\in P \end{array}$$

**Step 4.** Determining the vector of weights.

The vector of weights has the form $w=\left[w_{1},\ldots ,w_{n}\right]$, where

(19) $$w_{j}\in \left[0,1\right]\;\mathrm{and}\;\overset{n}{\underset{j=1}{\sum }}w_{j}=1.$$

The well-known objective method that can be applied in the intuitionistic fuzzy decision-making approach is the intuitionistic fuzzy entropy-based method (see Formulas (12)–(14)).

However, it should be noted that the weights can be determined by different methods. The objective weights used data from a decision matrix, while subjective weights are derived from the preferences defined by decision-makers (DMs). The most-used subjective methods include rank-ordering methods [59,60], SMARTER (SMART Exploiting Ranks) [61], DR (Direct Rating) [62,63], PA (Point Allocation) [64], and AHP (Analytic Hierarchy Process) [65], among others. 

**Step 5.** Calculating the weighted distances (*d_i_*^+^) between *i*-th alternative (*A_i_*) and the ideal-point (IFI^+^), using weighted Euclidean distance (see Formula (10)).

**Step 6.** Calculating the weighted distance (*d*^+−^) between the ideal point (IFI^+^) and anti-ideal point (IFI^−^), using weighted Euclidean distance (see Formula (10)).

**Step 7.** Calculating Double Intuitionistic Fuzzy value (DIFSM_i_) (for the *i*-th alternative:

(20) $${\mathrm{DIFSM}}_{i}=1-\frac{d_{i}{}^{+}}{d^{+-}}$$

**Step 8.** Ranking the alternatives according to the decreasing values of DIFSM.

It is also worth noting that the reference points can be determined by taking into account the largest and smallest intuitionistic fuzzy values, i.e.,

(21) $$\left(\mu_{j}^{+},\;\nu_{j}^{+}\right)=\{\begin{array}{c} \left(1,0\right)\;\mathrm{and}\;\pi_{j}^{+}=0\;if\;j\text{-}\mathrm{th}\;\mathrm{criterion}\in P\\ \left(0,1\right)\;\mathrm{and}\;\pi_{j}^{+}=0\;if\;j\text{-}\mathrm{th}\;\mathrm{criterion}\in N \end{array}$$

and

(22) $$(\mu_{j}^{-},\;\nu_{j}^{-})=\{\begin{array}{c} \left(1,0\right)\;\mathrm{and}\;\pi_{j}^{-}=0\;if\;j\text{-}\mathrm{th}\;\mathrm{criterion}\in N\\ \left(0,1\right)\;\mathrm{and}\;\pi_{j}^{-}=0\;if\;j\text{-}\mathrm{th}\;\mathrm{criterion}\in P \end{array}$$

Moreover, the weighted distance can be calculated using other formulas, for instance, the weighted Hamming measure (see Formula (11)).

## 5. Illustrative Example

In this section, to demonstrate the calculation process of the proposed approach, an example is provided. For comparison analysis, we adopt the example presented in [46]. 

A city is planning to build a municipal library. One of the problems facing the city development commissioner is to determine what kind of air-conditioning system should be installed in the library. The contractor offers five feasible alternatives, A_i_ (i = 1, 2, 3, 4, 5), that could be adapted to the physical structure of the library. Three criteria, C_1_ (economic), C_2_ (functional), and C_3_ (operational), are taken into consideration for the installation problem. All of them are benefit criteria. The weight vector of the weights is w = [0.3, 0.5, 0.2]. The decision matrix is presented in Table 1.

The proposed Double Intuitionistic Fuzzy Synthetic Measure method was applied to solve this problem with the vector of weights, w = [0.3, 0.5, 0.2], and with intuitionistic fuzzy entropy-based weights, calculated by Formulas (12)–(14). The entropy-based vector of weights obtained by Formulas (12)–(14) is as follows:

w = [0.1, 0.43, 0.47]

The intuitionistic fuzzy ideal point IFI^+^ based on max and min values (see Formula (17)) has the form:

$${\;\mathrm{IFI}}^{+}=[\left(0.8,\;0.2\right),\;\left(0.8,\;0),\;\left(0.9,\;0\right)\right].\;$$

The intuitionistic fuzzy anti-ideal point IFI^−^ based on max and min values (see Formula (18)) has the form:

$${\;\mathrm{IFAI}}^{-}=[\left(0.2,\;0.5\right),\;\left(0.5,\;0.2),\;\left(0.1,\;0.6\right)\right]$$

The distance between reference points determined by Formula (10) is the following:

$$d^{+-}=0.555$$

The distances between alternatives and the intuitionistic fuzzy ideal *d*^+^, DIFSM_i_ for two systems of weights are presented in Table 2.

Both the Hellwig method and TOPSIS are based on two reference points and the distances of alternatives from these points. The methods differ only in the aggregation procedure. Therefore, it seemed natural to compare the results obtained by both methods. We compared our results with the results obtained by the two variants of the weighted TOPSIS-based method proposed in the literature: (1) Weighted TOPSIS-based method with similarity measure (SMTOPSIS) [46] and (2) weighted intuitionistic fuzzy TOPSIS (IFTOPSIS). The SMTOPSIS and IFTOPSIS are defined as follows:

(23) $${\mathrm{SMTOPSIS}}_{i}=\frac{\;S_{i}{}^{+}}{S_{i}{}^{-}+S_{i}{}^{+}\;}=\frac{1-d_{i}{}^{+}}{\;1-d_{i}{}^{-}+1-d_{i}{}^{+}\;}$$

(24) $${\mathrm{IFTOPSIS}}_{i}=\frac{d_{i}{}^{-}}{\;d_{i}{}^{-}+d_{i}{}^{+}\;}$$

where $S_{i}{}^{+}=1-d_{i}{}^{+}$, $S_{i}{}^{-}=1-d_{i}{}^{-}$, $d_{i}{}^{+}$—the weighted distance between *i*-th alternative Ai and the ideal point $\left({\mathrm{IFI}}^{+}\right)$,$\;d_{i}{}^{-}$—the weighted distance between *i*-th alternative *A_i_* and the anti-ideal-point $\left({\mathrm{IFI}}^{-}\right)$, and $d_{i}{}^{+}\;\mathrm{and}\;d_{i}{}^{-}$ are calculated using Formula (10).

The values of the SMTOPSIS and IFTOPSIS measures along with the positions of the alternatives in the rankings are presented in Table 3 and Table 4.

The order of alternatives for the six combinations of measures and weight systems is presented in Table 5.

From Table 5, we can find that the ranking results obtained by these methods are slightly different. However, the best alternative is A_3_, which proves the feasibility and validity of the proposed methods. Based on Table 2, Table 3, Table 4 and Table 5, we further performed a detailed analysis in the following.

Firstly, by comparing sorting results for this same system of weights, we can observe differences with position alternatives A_5_ and A_1_ for the system of weights [0.3, 0.5, 0.2], and alternatives A_4_, A_2_ for the system of weights [0.1, 0.43, 0.47].

Secondly, the applied systems of weight in DIFSM did not affect the positions of the alternatives in the rankings. However, the values of the DIFSM measure for the entropy-based weights are characterized by greater dispersion than for the weights established in Xu [46].

Thirdly, in the case of the weights adopted for Xu [46], the method of constructing the SMTOPSIS and IFTOPSIS measures did not affect the ordering of the alternatives. Slight differences were only observed for the entropy-based weights. The same ordering of alternatives was obtained using the proposed measure DIFSM and IFTOPSIS (with weights determined based on the entropy measure).

**Figure 1:**
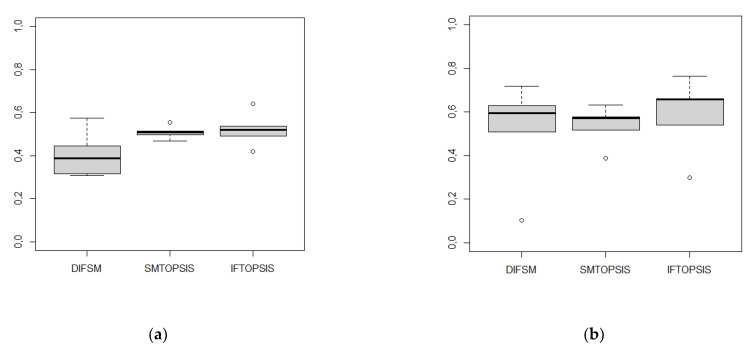
Box plots for DIFSM, SMTOPSIS, IFTOPSIS values. (**a**) system of weights w = [0.3, 0.5, 0.2] ([46]). (**b**) system of weights w = [0.1, 0.43, 0.47] (entropy-based).

The values of the measures for weights [46] (a) and based on entropy (b) are shown in Figure 1, respectively.

To sum up, according to the above comparative analysis, we can conclude in this example that the use of weights based on entropy definitely increased the value of the compared measures. In the case of the first system of weights, the proposed DIFSM measure differentiates the alternatives the most. The SMTOPSIS measure is characterized by the lowest discrimination of objects. The use of an entropy-based weighting system increased the discriminant ability of all comparable measures (especially SMTOPSIS and IFTOPSIS). In each of the three cases, the values of the measures for the best alternatives increased and the values for the worst ones decreased. The proposed DIFSM measure seems to be the least sensitive to the method of determining the weighting system presented in the study.

## 6. Conclusions

The article proposes the DISFM measure as an extension of the IFSM measure used to measure complex socio-economic phenomena based on ordinal data. To draw attention to the possibility of wider applications of both measures, the DIFSM algorithm was presented as a multi-criteria decision-making method.

The contributions of the paper are the following:

- We extended the Hellwig method into an intuitionistic fuzzy environment, showing the possible applications not only in the analysis of complex phenomena, but in a more general context of multi-criteria decision-making in uncertainty;
- The proposed aggregation formula DIFSM takes into consideration the different importance of criteria, and for dealing with the unknown information about criteria weights, the entropy-based weights of criteria methods were established;
- We adopt the Hellwig proposition of normalization of classical synthetic measure based on the distance between ideal and anti-ideal points into an intuitionistic fuzzy environment, which makes the DIFSM algorithm simpler and more intuitive than the approach based on average and standard deviations determined by the values of distances between alternative and ideal points.

The advantage of the proposed method is its simplicity and the possibility of applications in many research areas. The comparative analysis carried out in the article showed that the DIFSM measure may be characterized by greater discriminant abilities in relation to alternative measures. However, this requires confirmation through the analysis of larger data sets with different distributions of criteria assessments, because this article used a conventional example taken from the literature. Meanwhile, we can apply the proposed methods to deal with real-life multi-criteria problems, i.e., assessing socio-economic phenomena on the basis of survey data [29] or evaluations of negotiation offers [66].

Future research will also focus on assessing the sensitivity of the DIFSM measure to other weighting criteria systems available in the literature. The sensitivity of the DIFSM measure to other distance functions and other methods of establishing reference point coordinates will also be tested. Moreover, the authors plan to compare the usefulness and effectiveness of the DIFSM method with other multi-criteria methods proposed in a fuzzy intuitionistic environment.

## Figures and Tables

**Table 1:** Intuitionistic fuzzy decision matrix.

Alternative		C_1_		C_2_		C_3_
	*μ*	*ν*	*μ*	*ν*	*μ*	*ν*
A_1_	0.200	0.400	0.700	0.100	0.600	0.300
A_2_	0.400	0.200	0.500	0.200	0.800	0.100
A_3_	0.500	0.400	0.600	0.200	0.900	0.000
A_4_	0.300	0.500	0.800	0.100	0.700	0.200
A_5_	0.800	0.200	0.700	0.000	0.100	0.600

**Table 2:** Distances, ranks and DIFSM values.

System of Weights	w = [0.3, 0.5, 0.2]			w = [0.1, 0.43, 0.47]		
Alternative	*d* ^+^	DIFSM_i_	Rank	*d* ^+^	DIFSM_i_	Rank
A_1_	0.327	0.314	4	0.273	0.508	4
A_2_	0.292	0.389	3	0.225	0.594	3
A_3_	0.203	0.576	1	0.156	0.720	1
A_4_	0.265	0.445	2	0.205	0.630	2
A_5_	0.330	0.308	5	0.499	0.101	5

**Table 3:** Distances, ranks and SMTOPSIS values.

System of Weights	w = [0.3, 0.5, 0.2]				w = [0.1, 0.43, 0.47]			
Alternative	*d* ^+^	*d* ^−^	SMTOPSIS	Rank	*d* ^+^	*d* ^−^	SMTOPSIS	Rank
A_1_	0.673	0.763	0.469	5	0.727	0.679	0.517	4
A_2_	0.709	0.685	0.508	3	0.775	0.564	0.579	2
A_3_	0.798	0.639	0.555	1	0.844	0.494	0.631	1
A_4_	0.735	0.693	0.515	2	0.795	0.597	0.571	3
A_5_	0.670	0.682	0.495	4	0.501	0.790	0.388	5

**Table 4:** Distances, ranks and IFTOPSIS values.

System of Weights	w = [0.3, 0.5, 0.2]				w = [0.1, 0.43, 0.47]			
Alternative	*d* ^+^	*d* ^−^	IFTOPSIS	Rank	*d* ^+^	*d* ^−^	IFTOPSIS	Rank
A_1_	0.327	0.237	0.420	5	0.274	0.321	0.539	4
A_2_	0.292	0.315	0.519	3	0.226	0.436	0.658	3
A_3_	0.202	0.361	0.640	1	0.156	0.505	0.764	1
A_4_	0.265	0.307	0.537	2	0.206	0.403	0.661	2
A_5_	0.330	0.318	0.490	4	0.498	0.212	0.299	5

**Table 5:** Ranking alternatives based on the measures used.

Measure	System of Weights	Ranking Results
DIFSM	w = [0.3, 0.5, 0.2] [46]	A_3_ > A_4_ > A_2_ > A_1_ > A_5_
DIFSM	w = [0.1, 0.43, 0.47] entropy-based	A_3_ > A_4_ > A_2_ > A_1_ > A_5_
SMTOPSIS	w = [0.3, 0.5, 0.2] [46]	A_3_ > A_4_ > A_2_ > A_5_ > A_1_
SMTOPSIS	w = [0.1, 0.43, 0.47] entropy-based	A_3_ > A_2_ > A_4_ > A_1_ > A_5_
IFTOPSIS	w = [0.3, 0.5, 0.2] [46]	A_3_ > A_4_ > A_2_ > A_5_ > A_1_
IFTOPSIS	w = [0.1, 0.43, 0.47] entropy-based	A_3_ > A_4_ > A_2_ > A_1_ > A_5_

## Data Availability

Not applicable (for secondary data analysis, see [46]).

## Abbreviations

The following abbreviations are used in this manuscript:

DIFSM	Double Intuitionistic Fuzzy Synthetic Measure
IFS	Intuitionistic fuzzy sets
IFV	Intuitionistic fuzzy value
IFI	Intuitionistic fuzzy ideal point
IFAI	Intuitionistic fuzzy anti-ideal point
DM	Decision maker
DR	Direct Rating
PA	Point Allocation
AHP	Analytic Hierarchy Process
SMTOPSIS	Weighted TOPSIS-based method with a similarity measure
IFTOPSIS	Weighted intuitionistic fuzzy TOPSIS
TOPSIS	The Technique for Order of Preference by Similarity to Ideal Solution
